# PSYCHOMETRIC PROPERTIES OF THE DANISH VERSION OF THE CAREGIVER BURDEN SCALE: INVESTIGATING PREDICTORS AND SEVERITY OF BURDEN AFTER STROKE, SPINAL CORD INJURY, OR TRAUMATIC BRAIN INJURY

**DOI:** 10.2340/jrm.v56.34732

**Published:** 2024-05-02

**Authors:** Anne NORUP, Pernille Langer SOENDERGAARD, Mia Moth WOLFFBRANDT, Fin BIERING-SØRENSEN, Juan Carlos ARANGO-LASPRILLA, Frederik Lehman DORNONVILLE DE LA COUR

**Affiliations:** 1 Neurorehabilitation Research & Knowledge Centre, Copenhagen University Hospital – Rigshospitalet, Copenhagen, Glostrup; 2 Department of Neuroscience, University of Copenhagen, Copenhagen; 3 Neurorehabilitation-CPH, City of Copenhagen, Hellerup; 4 Department of Psychology, University of Southern Denmark, Odense; 5 Department of Clinical Medicine, University of Copenhagen, Copenhagen; 6 Department of Brain and Spinal Cord Injuries, Bodil Eskesen Centre, Rigshospitalet, Glostrup, Denmark; 7 Department of Psychology, Virginia Commonwealth University, Richmond, VA, USA; 8 The Elsass Foundation, Charlottenlund, Denmark

**Keywords:** caregiver burden, brain injuries, traumatic, predictors, spinal cord injuries, stroke

## Abstract

**Objective:**

To investigate (*i*) psychometric properties of the Danish version of the Caregiver Burden Scale, (*ii*) predictors of burden in caregivers of persons with stroke, spinal cord injury, or traumatic brain injury, and (*iii*) severity of caregiver burden, and compare level of severity of burden in caregivers of persons with stroke, spinal cord injury, or traumatic brain injury.

**Design:**

Cross-sectional study.

**Participants:**

Pooled sample of 122 caregivers.

**Methods:**

Psychometric properties including internal consistency, floor and ceiling effects, inter-item and item-total correlation were investigated using the Caregiver Burden Scale. Severity of burden was compared using Fisher’s exact test and ANOVA, and predictors of burden were investigated using multiple linear regression models.

**Results:**

The total burden score exhibited good internal consistency (α = 0.93), with no floor or ceiling effects. Longer time as a caregiver was a significant predictor of higher total score. The majority (52.2%) reported a low level of caregiver burden (below cut-off of 2.00). Mean scores on the Caregiver Burden Scale were not significantly different among caregivers across diagnostic groups. Differences were found when comparing spinal cord injury caregivers with brain injury caregivers (traumatic brain injury and stroke, collectively), χ^2^(2) = 6.38, *p* = 0.04, as spinal cord injury caregivers were more likely to report low levels of burden.

**Conclusion:**

Good psychometric properties were reported, and most caregivers reported a low level of burden, and longer time as a caregiver was associated with higher burden. Consequently, the Caregiver Burden Scale is a valid measure to use when measuring burden in caregivers of stroke, spinal cord injury, and traumatic brain injury patients.

LAY ABSTRACT

Acquiring an injury to the brain or spinal cord affects the person with the injury, but also the closest family. Very often close family members become informal caregivers providing daily care, which poses a significant burden. Caregiver burden among caregivers to persons with stroke, spinal cord injury, or traumatic brain injury were investigated, including the strengths and weaknesses of the Caregiver Burden Scale. The Danish version of the Caregiver Burden Scale was found to be valid. Longer time as a caregiver was associated with higher burden, and most caregivers reported a low level of burden.

After a neurological condition such as stroke, spinal cord injury (SCI), or traumatic brain injury (TBI), life is dramatically disrupted for the person concerned and their family members (1–3). Most of these individuals experience short and long-term physical, emotional, and cognitive sequelae that cause them to require some level of assistance to do everyday activities. Therefore, many family members become informal caregivers to support them with those tasks (4, 5). Most often, tasks for caregivers range widely and involve very different tasks such as nursing or caring tasks, practical tasks, or emotional tasks such as support etc. These tasks can pose a tremendous burden on the caregivers and closest family, and it is well established that caregiver burden has negative consequences on their physical and mental health (4, 6, 7). Adverse consequences have been reported such as long-term stress (8), decreased health-related quality of life (9–11), burnout (12), and symptoms of anxiety and depression (13, 14). In line with these results, recent studies have found that family members of individuals with SCI or TBI had higher healthcare use following the injury (3, 15).

The caregiver group is heterogeneous, and furthermore different consequences of stroke, SCI, and TBI might affect caregivers differently, resulting in diverse and complex needs (6). By investigating how different diagnoses affect caregivers, it is possible to pinpoint how more specific consequences (e.g. physical vs cognitive sequelae) affect caregivers and the burdens perceived. Consequently, such knowledge will shed light on patterns of burden related to different diagnoses and ultimately enable planning of interventions focusing on relief of specific burdens in caregivers. Furthermore, different studies have shown that caregiver burden increases over time (4), therefore it is very important that rehabilitation professionals have reliable instruments to assess burden in caregivers of individuals with the neurological conditions stroke, SCI, and TBI. This calls for a reliable and valid scale enabling use across diagnosis and caregiver populations, and no studies have examined and compared caregiver burden across stroke, SCI, and TBI.

The purpose of this study was to investigate: (*i*) the psychometric properties including internal consistency, floor and ceiling effects, inter-item and item-total correlation of the Danish version of the Caregiver Burden Scale (CBS); (*ii*) predictors of burden in caregivers of persons with stroke, SCI, or TBI; (*iii*) severity of caregiver burden and comparison of level of severity of burden in caregivers of persons with stroke, SCI, or TBI.

## METHODS

### Participants

The participants in the current study represent data collected across 2 different studies: an independent cohort (*n* = 44) of caregivers of persons with acquired brain injury (ABI; [16]) as well as a cohort (*n* = 84) of caregivers of people with stroke, SCI, or TBI included in a randomized controlled trial (RCT) (17). Caregivers in both trials were recruited from 2 highly specialized neurorehabilitation departments in the Eastern part of Denmark (Department of Brain Injuries and Department of Spinal Cord Injuries, both at Copenhagen University Hospital – Rigshospitalet). Both the ABI population and the SCI population included traumatic and non-traumatic injuries. The TBIs were caused by either a blow to the head or a penetrating injury, whereas the non-traumatic brain injuries (NTBI) were caused by, e.g., cerebrovascular diseases, ischaemic or haemorrhagic stroke, or infection (18). The traumatic SCI (tSCI) were caused by, e.g., falls, traffic accidents, sport accidents, or violence. In addition, the non-traumatic injuries (ntSCI) were caused by, e.g., infections, or degeneration and diseases with spinal stenosis or prolapsed discs (19). Individuals with ABI or SCI were primarily recruited from 2 highly specialized neurorehabilitation departments in the eastern part of Denmark. All caregivers included in the current study had to be caregivers of people with stroke, SCI, or TBI, consequently 4 caregivers from the original cohorts were excluded due to a different aetiology. All caregivers were 18 years or older at the time of inclusion and able to understand and speak Danish. Caregivers were excluded if they had previously been diagnosed with any neurologic or psychiatric disorder, or had a history of violence or substance abuse in their family.

### Study design

The first caregiver cohort was recruited as a part of a follow-up study investigating caregiver needs and burden in the chronic phase following severe ABI (16, 20). The second cohort was part of the RCT described above, where data from the baseline assessment before randomization were used (20). All participants provided written informed consent in accordance with the Helsinki Declaration. The 2 studies were approved by the Danish Data Protection Agency (journal no. P-2021-603 & 2013-41-2007) and the Committees on Health Research Ethics on the Capital Region of Denmark (journal no. H-1801 4858). The RCT was registered on 24 January 2019 at ClinicalTrials.gov, identifier: NCT03814876, where the protocol, 2018_0004, Family Intervention Following Traumatic Injury, is accessible.

### Measures

Sociodemographic data (age, gender, work, marital, and relationship status) were collected from each caregiver and care recipient at time of inclusion. Furthermore, data concerning caregiver role were collected when the caregiver was included, and these included the socio-demographic data mentioned earlier as well as time in caregiver role. Length of stay in hospital and other injury-related measures were collected from the care recipient’s hospital file. All data were collected when care recipient and caregiver were included in the study. Caregiver burden was assessed using the CBS (21), which has been developed in Sweden. The CBS was developed by factor analysis and designed to be valid regardless of diagnosis. The CBS has been used to measure burden among caregivers to persons with various diagnoses such as stroke (21), dementia (22), TBI (23), ABI (16), and long-term illness and/or old age (24). The CBS consists of 22 questions scored from 1–4 with 4 response options: “not at all”, “seldom”, “sometimes”, and “often”. The CBS is divided into 5 factors commonly referred to as sub-scales: General Strain (8 questions), Disappointment (5 questions), Emotional Involvement (3 questions), Environment (3 questions) and Isolation (3 questions). A Total Burden index is given by calculating the mean of all 22 items ranging from 1 to 4, where higher score indicates greater burden. The items cover aspects such as caregiver health, psychological well-being, relationship, social network, physical workload, and environmental aspects, with statements such as “Do you feel tired and worn out?”, “Do you ever feel offended and angry with your relative?”, and “Do you think your own health has suffered because you have been taking care of your relative?”.

The overall mean caregiver burden was divided into 3 groups: low burden (1.00–1.99), medium burden (2.00–2.99), and high burden (3.00–4.00), as suggested by Andrén and Elmståhl (25). The CBS has previously demonstrated satisfactory validity and reliability with kappa values in the range of 0.89–1.0 in a population of stroke patients (21).

### Statistical analyses

*Psychometric properties*. Internal consistency was evaluated using Cronbach’s alpha, and values between 0.70 and 0.95 were considered to be adequate (26). Floor and ceiling effects were evaluated using frequencies of the lowest and highest possible scores, respectively. Frequencies ≥ 15% was used to indicate a floor or ceiling effect (26). Individual items were evaluated using descriptive item statistics, including means, SD, inter-item correlations, and item-total correlations, corrected for item overlap. Average inter-item correlation and item-total correlations ≥ 0.30 were considered to be adequate (27). All correlations were conducted using Spearman’s rank-order correlation coefficient. All statistics were computed for the total score and each subscale score in the total group and by type of injury.

Predictors of burden were examined using multiple linear regression models. One model was specified for each subscale and the total score of CBS. Three categorical and 3 continuous predictors were investigated simultaneously for each subscale and the total scale, including type of injury (stroke, SCI, or TBI), type of relationship (spouse, parent, or other), gender (male or female) and age of the caregiver, length of stay in hospital, and longer time in caregiver role. For type of relationship, the levels “Child”, “Sibling”, and “Other” were collapsed into “Other” due to few observations (< 5%). In initial data analysis, length of stay in hospital and time in caregiver role were log2 transformed to deal with substantial skewness and univariate outliers. Transformation improved the distribution of regression residuals in respect of the assumptions of linearity, normality, and homoscedasticity. No multicollinearity was indicated (all generalized variation-inflation factors were < 1.6). In primary analysis, missing data were handled using listwise deletion. A sensitivity analysis was conducted to examine the influence of missing data using multiple imputation, as ≥ 5% of cases had missing data. Multiple imputation was conducted using fully conditional specification applied to all the variables in all regression models, including all CBS scale scores as auxiliary variables. The number of imputed datasets was determined by the percentage of cases with missing data (18%).

Severity of caregiver burden was estimated by calculating unadjusted means and frequencies of burden categories. Severity of burden was compared across injury groups using Fisher’s exact test (for burden categories) and ANOVA test (for mean scores). Analyses were completed comparing effects across groups (e.g. TBI vs stroke vs SCI), but furthermore, the two groups of ABI, namely TBI and stroke were collapsed and collectively compared with SCI. These analyses were completed as the consequences of SCI and ABI are different and could affect burden dissimilarly.

Analyses were conducted in R version 4.2.0 (28) using base *lm* function and the *car* package for regression analysis (29) and *mice* for multiple imputation (30).

## RESULTS

The study included a total of 233 participants, respectively 122 caregivers and 111 care recipients. The sample consisted of 40 caregivers of individuals with stroke, 24 caregivers of individuals with SCI, and 58 caregivers of individuals with TBI. The caregivers had a mean age of 52 ± 14.4 years and were predominantly female (66.4%). The majority were spouses or partners (55.7%) or parents (23.0%) of the person with the injury. Caregivers had provided care for 33 months on average. The care recipients had a mean age of 52.5± 15.2 years and were predominantly male (65.8%). Mean length of stay in hospital was 101.8 ± 69.9 days. Socio-demographics are described in Table I.

**Table I:** Characteristics of patients with spinal cord injury, traumatic brain injury and stroke and their caregivers

Variable	Spinal cord injury (*n* = 24)	Traumatic brain injury (*n* = 58)	Stroke (*n* = 40)	Total (*n* = 122)
*Caregivers*				
Gender, *n* (%)				
Male	8 (33.3)	14 (24.1)	19 (47.5)	41 (33.6)
Female	16 (66.7)	44 (75.9)	21 (52.5)	81 (66.4)
Age, years, mean (SD)	51.9 (17.3)	54.1 (13.1)	48.6 (14.1)	52.0 (14.4)
Relationship to care recipient, *n* (%)				
Spouse or partner	15 (62.5)	30 (51.7)	23 (57.5)	68 (55.7)
Parent	4 (16.7)	20 (34.5)	4 (10.0)	28 (23.0)
Child	2 (8.3)	4 (6.9)	10 (25.0)	16 (13.1)
Sibling	2 (8.3)	1 (1.7)	2 (5.0)	5 (4.1)
Other	1 (4.2)	3 (5.2)	1 (2.5)	5 (4.1)
Marital status, *n* (%)				
Married or cohabiting with partner	21 (91.3)	45 (77.6)	30 (75.0)	96 (79.3)
Partner, not cohabiting	1 (4.2)	3 (5.2)	1 (2.5)	5 (4.1)
Single	1 (4.2)	6 (10.3)	6 (15.0)	13 (10.7)
Other	0	4 (6.9)	3 (7.5)	7 (5.8)
Work status, *n* (%)				
Full-time occupation	12 (50.0)	34 (58.6)	29 (72.5)	75 (61.5)
Student	4 (16.7)	2 (3.4)	2 (5.0)	8 (6.6)
Homemaker	0	2 (3.4)	1 (2.5)	3 (2.5)
Unemployed	1 (4.2)	2 (3.4)	2 (5.0)	5 (4.1)
Retired	5 (20.8)	15 (25.8)	6 (15.0)	26 (21.3)
Sick leave	2 (8.3)	1 (1.7)	0	3 (2.5)
Missing	0	2 (3.4)	0	2 (1.5)
Time in caregiver role, months, mean (SD)	19.7 (12.2)	42.3 (28.8)	26.3 (23.9)	33.2 (26.6)
Median (IQR)	18.0 (6.75–28.75)	47.0 (12.0–67.0)	17.0 (11.0–47.2)	21.0 (11.5–51.5)
Care recipients, *n* (%)	20 (18.0)	57 (34.8)	34 (36.2)	111 (100)
Age of care recipient, years, mean (SD)	48.4 (17.6)	53.1 (15.9)	54.1 (12.2)	52.5 (15.2)
Sex of care-recipients, *n* (%)				
Male	14 (70.0)	41 (71.9)	18 (52.9)	73 (65.8)
Female	6 (30.0)	16 (28.1)	16 (47.1)	38 (34.2)
Length of stay in hospital, days, mean (SD)	160.2 (114.3)	85.5 (46.7)	91.4 (36.8)	101.8 (69.9)
Median (IQR)	113.5 (80.0–220.0)	80.0 (50.0–110.5)	88.0 (68.5–103.5)	84.5 (62.5–122.5)

SD: standard deviation; IQR: interquartile range.

### Psychometric properties and summary statistics of the Caregiver Burden Scale

The total burden score exhibited good internal consistency, α = 0.93, and no floor or ceiling effects (Table II), indicating good psychometric properties.

**Table II:** Mean scores, internal consistency, and floor and ceiling effects of the Caregiver Burden Scale for caregivers of patients with spinal cord injury, traumatic brain injury and stroke

Factor	Valid, *n*	*M* (SD)	Skew	Floor, %	Ceiling, %	α
Total group *(n* = 122)						
Total burden	113	2.05 (0.64)	0.51	1.64	0.00	0.93
General strain	118	2.28 (0.79)	0.28	4.10	0.82	0.90
Isolation	119	1.91 (0.85)	0.71	25.41	2.46	0.75
Disappointment	119	2.12 (0.73)	0.29	7.38	0.82	0.76
Emotional involvement	121	1.96 (0.73)	0.94	8.20	3.28	0.78
Environment	120	1.67 (0.67)	0.96	30.33	0.00	0.62
Caregivers, persons with SCI (*n* = 24)						
Total burden	23	1.85 (0.47)	0.80	0.00	0.00	0.89
General strain	23	2.03 (0.59)	0.41	4.35	0.00	0.80
Isolation	24	1.62 (0.60)	0.61	29.17	0.00	0.58
Disappointment	24	1.92 (0.64)	0.32	12.50	0.00	0.75
Emotional involvement	24	1.74 (0.53)	1.61	0.00	0.00	0.79
Environment	24	1.56 (0.60)	0.79	37.50	0.00	0.59
Caregivers, persons with TBI (*n* = 58)						
Total burden	52	2.13 (0.72)	0.39	3.85	0.00	0.95
General strain	56	2.38 (0.88)	0.16	5.36	1.79	0.92
Isolation	55	2.01 (1.00)	0.66	25.45	5.45	0.87
Disappointment	55	2.20 (0.77)	0.10	9.09	0.00	0.78
Emotional involvement	57	2.12 (0.79)	0.41	12.28	3.51	0.77
Environment	57	1.63 (0.68)	1.08	33.33	0.00	0.63
Caregivers, persons with stroke (*n* = 40)						
Total burden	38	2.06 (0.61)	0.24	0.00	0.00	0.93
General strain	39	2.28 (0.74)	0.07	2.56	0.00	0.90
Isolation	40	1.94 (0.74)	0.15	25.00	0.00	0.58
Disappointment	40	2.14 (0.72)	0.42	2.50	2.50	0.73
Emotional involvement	40	1.87 (0.71)	1.43	7.50	5.00	0.78
Environment	39	1.79 (0.69)	0.78	23.08	0.00	0.59

SCI: spinal cord injury; TBI: traumatic brain injury.

Among the subscales, the General Strain, Disappointment, and Emotional Involvement subscales exhibited adequate properties (Table II) indicated by internal consistency and no floor or ceiling effects. However, internal consistency was poor for the 3 items on the Environment subscale, α = 0.62. In addition, both the Environment and Isolation subscales exhibited considerable floor effects, 30.3% and 25.4%, respectively.

Notably, the Isolation subscale performed differently across injury groups. Internal consistency was adequate in the TBI caregiver subgroup, α = 87, but poor in the SCI and stroke caregiver subgroups, both α = 0.58.

All items were positively inter-correlated within subscales; however, 2 items were weakly correlated with the other items within their respective subscales, namely Item 18 (Disappointment) and Item 15 (Environment) (see Table SI). Three additional items on the Environment (Item 9) and Emotional Involvement (Item 11 and 16) subscales exhibited weak average correlations among all 22 items, despite being correlated within their respective subscales (see Table SI).

### Predictors of caregiver burden

**Fig. 1:**
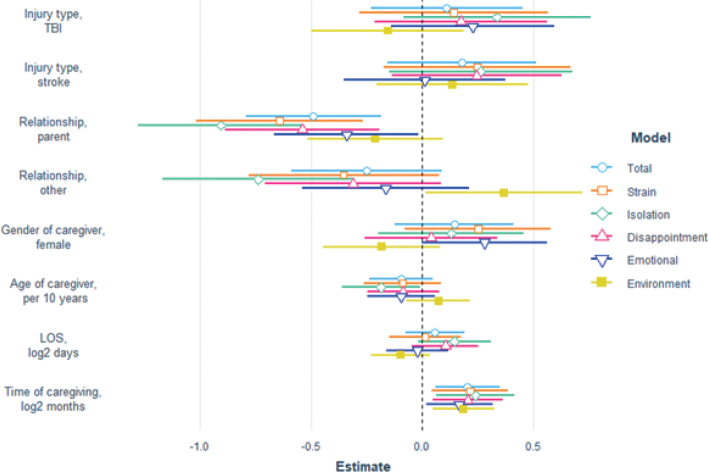
**Predictors of caregiver burden.** The figure illustrates a multiple linear regression model, where the figure depicts standardized regression coefficients and 95% confidence intervals. For categorical predictors, the reference value was male spouse to a care recipient with spinal cord injury. TBI: traumatic brain injury; LOS: length of stay.

In multiple linear regression, 18% to 31% of the variation between individuals in CBS scores was explained by the models. All regression models were statistically significant, all *p* ≤ 0.01 (see Table SII). Time in caregiver role was a significant predictor of the total score and all subscale scores on CBS (Fig. 1). For the total score, double the months of caregiving since injury onset was independently associated with an increased burden of 0.16 points (95% CI [0.05, 0.28], *p* = 0.005). In the sample, time of caregiving ranged from 6 to 93 months (almost 8 years). Thus, the longest time of caregiving was almost 16 times longer than the shortest, which is equal to a log2 fold difference of 4 (doubling from 6, 4 times). This means that a caregiver who has provided care for 93 months is expected to score almost 0.16 × 4 = 0.64 points more in total burden compared with someone who has provided care for 6 months. That is equal to one standard deviation in this sample.

The type of relationship to the person with injury was also a significant predictor. Being a spouse or partner was associated with a greater burden compared with being a parent of the care recipient on all scores but the Environment subscale (Fig. 1). In addition, partners were also predicted to score significantly higher on the Isolation subscale compared with other types of relationships such as children or siblings (B = 0.74, 95% CI [0.31, 1.17], *p* = 0.001), but lower on the Environment subscale (B = –0.37, 95% CI [–0.72, –0.02], *p* = 0.04). Finally, younger caregivers were predicted to report higher level of burden in terms of Isolation (B = –0.13 per 10 years of age, 95% CI [–0.25, –0.01], *p* = 0.04), and female caregivers report more burden than male caregivers in terms of Emotional Involvement (B = 0.28, 95% CI [0.00, 0.56], *p* = 0.05). Length of stay in hospital and the type of injury did not significantly predict subsequent perceived burden of caregivers (Fig. 1).

### Sensitivity analysis

Notably, between 11.5% and 18.0% of cases were omitted in regression analyses due to missing data. Using multiple imputation, the results differed substantially for the regression on the Environment score. The model explained less variance (12%), F_pooled_(8,113) = 1.85, *p* = 0.08, and none of the predictors were statistically significant for this subscale. The results on the other CBS scores remained robust (Table SII and Table SIII).

### Severity and comparison of level of burden

Mean scores on the total scale and each subscale of the CBS were not significantly different among all 3 injury groups (Table II). In Table III, severity of caregiver burden is reported. The majority (52.2%) of the full sample reported a low level of caregiver burden (< 2.00), and few (8.8%) reported high levels of burden (≥ 3.00). Almost 40% reported medium levels of burden. Differences were found when comparing SCI with ABI (TBI and stroke, collectively), χ^2^(2) = 6.38, *p* = 0.04, as caregivers to individuals with SCI were more likely to report low levels of caregiver burden (standardized residual = 2.33). Proportions did not differ significantly among all three groups, χ^2^(4) = 7.65, *p* = 0.12.

**Table III:** Number and percentage of caregivers of patients with spinal cord injury, traumatic brain injury, and stroke with low, medium, or high burden

Factor	Low (1.00–1.99)	Medium (2.00–2.99)	High (3.00–4.00)	*n*
Spinal cord injury	17 (73.9%)	6 (26.1%)	0	23
Traumatic brain injury	25 (48.0%)	20 (38.5%)	7 (13.5%)	52
Stroke	17 (44.7%)	18 (47.4%)	3 (7.9%)	38
Total group	59 (52.2%)	44 (39.0%)	10 (8.8%)	113

## DISCUSSION

The Danish version of the CBS revealed that the total burden score, and the 3 scales General Strain, Disappointment, and Emotional Involvement, had adequate internal consistency and no floor or ceiling effects among caregivers to people with stroke, TBI, and SCI. In the total sample, most caregivers scored a low burden; however, a fairly large proportion scored as indicating a medium burden. Significantly lower burden was seen in caregivers of persons with SCI. In the total group of caregivers, longer time in the caregiver role or being a spouse or partner predicted higher burden.

### Psychometric properties of the Danish version of the CBS

The first aim was to evaluate internal consistency, floor and ceiling effects, inter-item and item-total correlation of the CBS in samples of caregivers to persons with stroke, SCI, or TBI. The CBS comprises 6 composite scores, including the total score of all 22 items (total burden) addressing distinct aspects of burden. The total burden score and 3 subscale scores, General Strain, Disappointment, and Emotional Involvement, exhibited adequate internal consistency and no floor or ceiling effects across and within each injury group. The Environment subscale exhibited inadequate internal consistency and floor effects in all samples, which has been found before in previous studies (21). Consequently, scores on this subscale should be interpreted with caution when used for caregivers to adults with brain or spinal cord injury. This subscale comprises 3 items only and is thus more susceptible to unreliable results compared with larger scales with more items. The Isolation subscale also had floor effects, and internal consistency was inadequate for caregivers to persons with stroke or SCI, but not TBI, indicating that the items of this subscale may only indicate burden in respect of isolation reliably among TBI caregivers. Thus, the type of injury may be important to how caregivers respond to the items on the Isolation subscale. Specifically, behaviour change and lack of inhibition are reported after TBI, but not after SCI. Such specific consequences of injury might affect primarily TBI caregivers, as measured by the Isolation subscale on CBS.

### Predictors of burden

In the current study, higher burden on all subscales and total burden were predicted by longer time in caregiver role, which is in accordance with previous studies within ABI (16, 23, 31, 32). The study by Jaracz et al. had a longitudinal design up to 5 years post injury, and their results indicated that the association between hours spent caregiving were still evident 5 years after injury (32). However, one TBI study conducted within the severe TBI group found no association with time in caregiver role and burden conducted 3 months to 120 months after onset of injury (33) using the Zarit Burden Interview. One SCI study found no change in burden over time measured with the Caregiver Strain Index (34). Altogether, the evidence within ABI does suggest that time in caregiver role is associated with increased burden.

Another significant predictor in our study was being spouse or partner, which was associated with higher burden on all scales expect the Environment scale. This has been reported previously in other studies investigating effects of ABI (16, 35, 36). However, a few studies have reported no association between being a partner or spouse and increased burden (32, 37, 38), or increased burden in caregivers living with the care recipient (39). We did find predictors solely related to the subscales Isolation and Emotional Involvement. In our sample younger caregivers perceived greater burden on the Isolation subscale. Importantly, this association with age was independent of the caregiver relationship, i.e., being a parent or partner, etc. This effect of age has been reported only once previously, namely by Kruithof et al. (40) investigating a group of caregivers 2 months after stroke. The opposite association was found in an SCI study, where higher caregiver strain scores were reported by older caregivers (34). This might support the fact that the caregiver role is significantly different across diagnostic groups, as the physical need for help might be more severe in the SCI group, which might be more demanding for older caregivers, whereas an ABI in a young family will inevitably affect the whole family, as in many cases the deficits might also be cognitive or behavioural. However, most studies have not found any associations between caregiver age and burden (32, 33, 35, 38, 41).

On the Emotional Involvement subscale, we found that female caregivers reported higher levels of burden. This has also been reported in an SCI group previously (2), but the opposite pattern has also been reported among stroke caregivers, where males reported higher burden (41). But as already mentioned, most studies have found no association between gender and burden (23, 32, 35, 38, 42, 43).

### Severity and comparison of level of burden

In the total sample, the majority scored a low burden, but a relatively large proportion scored a medium burden. As far as the authors are aware, no other studies have reported on caregiver burden across aetiology and, consequently, severity can primarily be compared with similar diagnostic groups. The TBI numbers are comparable to the ones reported by Manskow et al. (35) in TBI caregivers, where caregiver burden was reported as high in 16% of caregivers and moderate in 34%, comparable to a high burden of 13.5% and moderate burden of 38.5% in our study. The mean total burden index in the study by Manskow et al. was 2.12 (35), whereas the comparable number in our study was 2.13. A recent study has reported a higher of burden among stroke caregivers corresponding to 21%, but in this study responses were dichotomized and no group of medium burden was reported, which makes comparison difficult (24).

In general, SCI caregivers were more likely to report a low level of caregiver burden, as all median values were below 2.00 in the SCI caregiver group. It has previously been reported that the majority of family caregivers of persons with SCI cope well with their new life situation (44), and this might underline how the situation of the families affected by ABI is significantly different from the situation of families living with SCI. However, research in this area is scant, and very few studies have compared different groups of caregivers. Very early studies did point to the fact that TBI caregivers reported higher levels of stress than SCI caregivers, and indicated the association with personality change in persons with TBI as a potential explanation (45, 46). Similar results have been reported by Anderson et al. (47), where caregivers of persons with TBI reported a higher burden, although non-significant, than SCI caregivers at 2 and 5 years post injury (47).

### Strengths and limitations

This is the first study to address and compare caregiver burden across different neurological conditions. Consequently, a strength of this study is the inclusion of caregivers providing care for persons living with sequelae of different neurological diagnoses. Another strength is the detailed evaluation of internal consistency and floor and ceiling effects of the CBS, which, to the authors’ knowledge, has not been carried out before in the Danish version.

Even though this study evaluated important psychometric properties, several properties were not evaluated, e.g., in relation to construct validity or factorial validity. Thus, these results do not indicate the extent to which the scale scores represent caregiver burden or other constructs, or whether the current subsets of items reflect the dimensional structure of caregiver burden. Furthermore, the limited sample size in each of the injury groups might lead to less accurate results and affects the generalisability of the results.

Another limitation is the time span when the sample was collected, which could bias the results. A subgroup of the caregivers might have been affected by the COVID-19 pandemic, as they were recruited during the pandemic, and this could influence the burden experienced.

No similar measure indicating severity of injury was available across the 3 different diagnostic groups, but all patients were treated in highly specialized units receiving patients with primarily severe injuries in terms of both ABI and SCI. However, a measure of functional independence or similar would have provided valuable information on the possible association between functional level and caregiver burden feedback.

The CBS is a self-report measure and does not provide an objective measure of burden. Furthermore, the study had a cross-sectional design. Consequently, we have no information concerning caregiver burden across time. Similarly, we did not have the opportunity to investigate whether the psychometric properties of the scale change over time, or if associations between predictors of burden and perceived burden change over time. Future studies using invariance analyses should be done in order to confirm this.

### Conclusion

The study supports the use of the Danish version of CBS in stroke, SCI, and TBI caregivers, with good internal consistency and no floor or ceiling effects for the total score and General Strain, Disappointment, and Emotional Involvement. Consequently, CBS is a valid measure to use when measuring burden in caregivers of stroke, SCI, and TBI. Most caregivers reported a low level of burden. Longer time in caregiver role and being a spouse or partner of the care recipient was associated with higher burden. Future research should investigate the differences between burden reported by caregivers in different diagnostic groups, as associations between perceived burden in caregivers and deficits in the care recipient are still sparsely investigated.

## Supplementary Material

PSYCHOMETRIC PROPERTIES OF THE DANISH VERSION OF THE CAREGIVER BURDEN SCALE: INVESTIGATING PREDICTORS AND SEVERITY OF BURDEN AFTER STROKE, SPINAL CORD INJURY, OR TRAUMATIC BRAIN INJURY

PSYCHOMETRIC PROPERTIES OF THE DANISH VERSION OF THE CAREGIVER BURDEN SCALE: INVESTIGATING PREDICTORS AND SEVERITY OF BURDEN AFTER STROKE, SPINAL CORD INJURY, OR TRAUMATIC BRAIN INJURY
