# Supplementary material for: PSYCHOMETRIC PROPERTIES OF THE DANISH VERSION OF THE CAREGIVER BURDEN SCALE: INVESTIGATING PREDICTORS AND SEVERITY OF BURDEN AFTER STROKE, SPINAL CORD INJURY, OR TRAUMATIC BRAIN INJURY
Source: J Rehabil Med. 2024 May 2;56:34732. doi: 10.2340/jrm.v56.34732 (PMC11091905; doi:10.2340/jrm.v56.34732)
Supplement: PSYCHOMETRIC PROPERTIES OF THE DANISH VERSION OF THE CAREGIVER BURDEN SCALE: INVESTIGATING PREDICTORS AND SEVERITY OF BURDEN AFTER STROKE, SPINAL CORD INJURY, OR TRAUMATIC BRAIN INJURY [file JRM-56-34732-s1.pdf]

Supplementary material has been published as submitted. It has not been copyedited, or typeset by Journal of Rehabilitation Medicine

Fig. S1. Scale score distributions in the total group (N = 122)

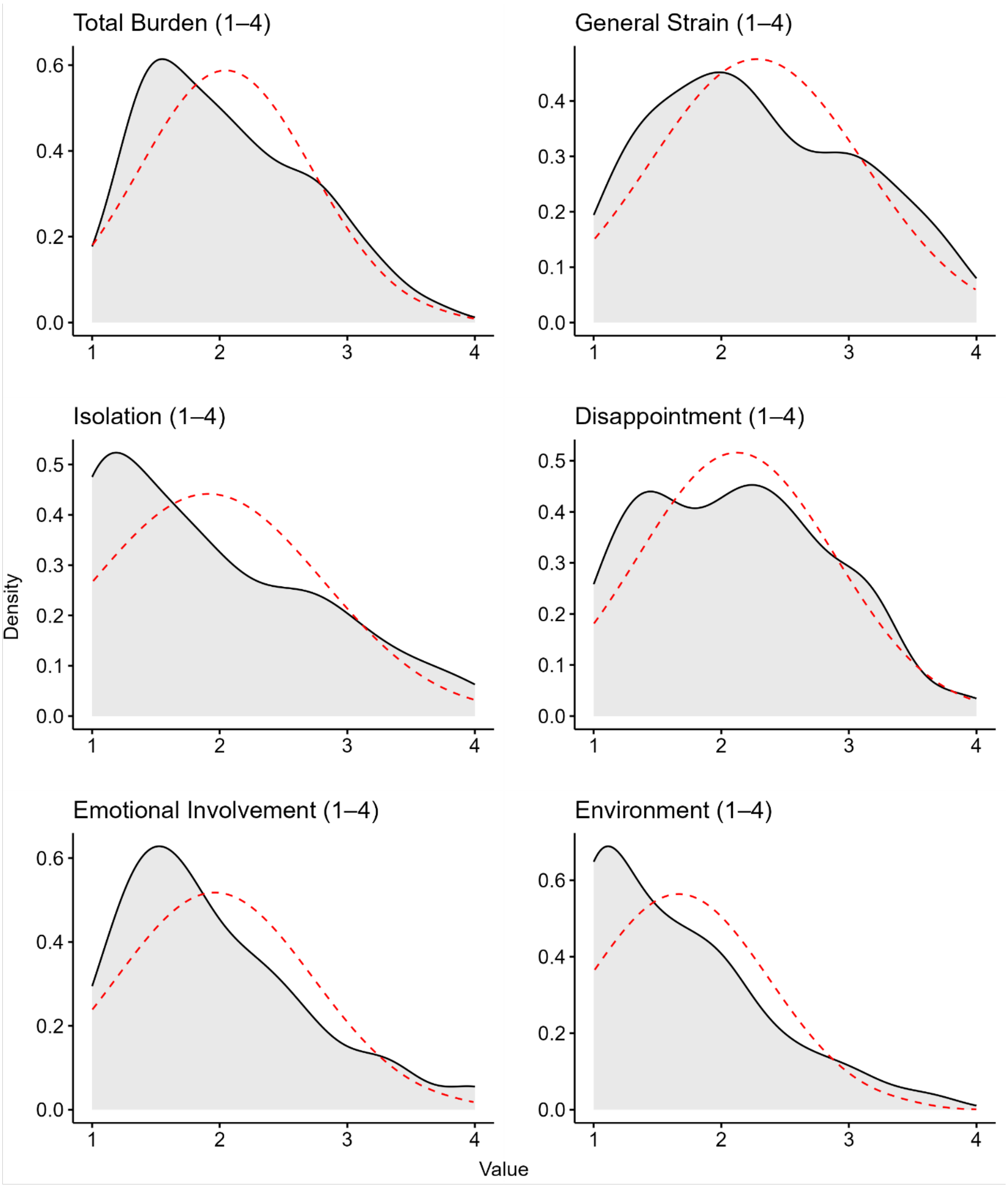

Note. The black solid line depicts the density curve of observed values. The red dashed line depicts the normal distribution. The Total Burden, General Strain, and Disappointment scales exhibited approximately symmetric distributions. The Isolation and Environment subscales exhibited floor effects and a moderate positive skew. The Emotional Involvement exhibited a moderate positive skew despite no floor effects.
